# The Effect of Small Molecule Pharmacological Agents on the Triterpenoid Saponin Induced Endolysosomal Escape of Saporin and a Saporin-Based Immunotoxin in Target Human Lymphoma Cells

**DOI:** 10.3390/biomedicines9030300

**Published:** 2021-03-15

**Authors:** Harrison J. Wensley, Wendy S. Smith, Suzanne E. Holmes, Sopsamorn U. Flavell, David J. Flavell

**Affiliations:** 1 Clinical and Experimental Sciences, Faculty of Medicine, University of Southampton, Southampton General Hospital, Southampton SO16 6YD, UK; harrison.wensley@googlemail.com; 2 The Simon Flavell Leukaemia Research Laboratory, Southampton General Hospital, Southampton SO16 6YD, UK; WendyS@leukaemiabusters.org.uk (W.S.S.); SuzanneH@leukaemiabusters.org.uk (S.E.H.); BeeF@leukaemiabusters.org.uk (S.U.F.); 3 Faculty of Medicine, University of Southampton, Southampton General Hospital, Southampton SO16 6YD, UK

**Keywords:** saporin, immunotoxin, augmentation, saponin, endocytosis

## Abstract

Triterpenoid saponins augment the cytotoxicity of saporin based immunotoxins. It is postulated that this results from a saponin-mediated increase in the endolysosomal escape of the toxin to the cytosol, but this remains to be confirmed. To address this issue, we used a number of pharmacological inhibitors of endocytic processes as probes to investigate the role played by saponin in the endolysosomal escape of fluorescently labeled saporin and a saporin based immunotoxin targeted against CD38 on human lymphoma and leukemia cell lines. Endolysosomal escape of the toxin was measured by flow cytometric pulse shape analysis. These results were compared to the effects of the various inhibitors on the saponin-mediated augmentation of toxin and immunotoxin cytotoxicity. Inhibitors of clathrin-mediated endocytosis, micropinocytosis, and endosomal acidification abrogated the saponin-induced increase in the endolysosomal escape of the toxin into the cytosol, suggesting that these processes may be involved in the internalization of saponin to the same endolysosomal vesicle as the toxin. Alternatively, these processes may play a direct role in the mechanism by which saponin promotes toxin escape from the endolysosomal compartment to the cytosol. Correlation with the effects of these inhibitors on the augmentation of cytotoxicity provides additional evidence that endolysosomal escape is involved in driving augmentation.

## 1. Introduction

Targeted toxins are chimeric therapeutic agents that consist of a protein toxin molecule possessing a cytotoxic enzymatic activity coupled to a tumor specific cell binding domain such as a cytokine, growth factor, or antibody (immunotoxin). The clinical development of these agents has been hindered by their immunogenicity [1] and the development of dose-limiting toxicities that include vascular leak syndrome and hepatotoxicity thought to result of nonspecific uptake by off-target bystander cells [2,3,4]. Because of these hurdles, only two targeted toxins have thus far been approved by the FDA for the treatment of malignancy [5,6]. Widening the therapeutic window of targeted toxins so that a useful therapeutic effect can be achieved at lower dose levels would represent a major step forward for the clinical development of this class of targeted therapeutic molecule.

The ribosome inactivating protein (RIP) saporin [7] from Saponaria officinalis (common soapwort) is an excellent candidate for the toxin component of targeted toxins. It is highly stable, being resistant to high temperatures and proteolysis [8], and the chemical modifications used in the conjugation process to incorporate it into a targeted toxin do not significantly affect its catalytic activity [9]. Furthermore, saporin is a 30kDa single chain type I RIP that does not possess a cell binding domain [10,11]. It therefore lacks an efficient means of cell entry, making it significantly less cytotoxic in its unconjugated form than type II RIPs such as intact ricin. This should in principle reduce the off-target effects of saporin-based targeted toxins. Type I RIPs such as saporin possess N-glycosidase activity (EC 3.2.2.22), whose action is to depurinate the 60S ribosomal subunit and prevent its interaction with the elongation factor EF-2 thereby irreversibly blocking translation [12] and arresting protein synthesis. To exert this activity, RIPs such as saporin must enter the cytosol of target cells in order to gain access to the ribosome. Poor endolysosomal escape of saporin-based targeted toxins to the cytosol has been identified as a major factor limiting their efficacy, with internalized toxin being trafficked to the lysosome where it undergoes subsequent degradation [13,14].

Saponins, membrane permeabilizing, plant derived aglycones have been shown to augment the cytotoxicity of saporin and saporin-based targeted toxins at sub-toxic concentrations [15,16,17]. Confocal microscopy studies utilizing fluorescently labeled saporin and saporin based immunotoxins (ITs) have provided evidence suggesting that saponins increase the endolysosomal escape of these toxins into the cytosol [17,18]. The mechanism by which saponins enhance endolysosomal escape has not been fully elucidated. We and others have hypothesized that saponin is internalized and co-localizes to the same endolysosomal compartment as saporin [16]. Here, the saponin facilitates the endolysosomal escape of the saporin into the cytosol via a currently unknown mechanism. This may occur due to an accumulation and subsequent concentration of saponin in the endolysosomal lumen so that a permeabilizing concentration is achieved within the luminal space.

Experimental evidence has shown that the enhancement of the endolysosomal escape of saporin by saponin is dependent on an acidic pH in the endolysosomal lumen. Inhibitors of endosomal acidification abrogate the augmentation of saporin cytotoxicity by saponins [18,19]. They have also been shown to prevent the saponin induced endolysosomal escape of an Alexa Fluor conjugated saporin toxin and saporin based IT [13,18].

The proposed co-localization hypothesis requires that both saporin and saponin are trafficked to the same endolysosomal vesicle. The internalization and trafficking route of various saponin species has not yet been determined, and it is not currently known whether saponins are internalized from the fluid phase via an endocytic process or alternatively following their integration into portions of the plasma membrane that are subsequently endocytosed. Smith et al. recently investigated the effects of a range of inhibitory small molecule pharmacological agents on the augmentation of saporin and a number of saporin based ITs by saponinum album (SA), a mixture of triterpenoid saponins derived from Gypsophila species [19]. These experiments aimed to determine the role played in the augmentation of cytotoxicity by endocytic processes such as clathrin mediated endocytosis and macropinocytosis and by the polymerization of actin and microtubules, which are involved in the endocytosis and the trafficking of endocytic vesicles, respectively. The process of endosomal acidification was also inhibited by two different pharmacological inhibitors of acidification each with a different mechanism of action. The findings of Smith and co-workers revealed that the augmentation of saporin based ITs by SA was abrogated by a number of the pharmacological agents investigated, yielding clues as to the mechanism(s) behind saponin-induced augmentation effects on targeted toxins.

In the study described here, we aimed to explore the effect of the same range of pharmacological inhibitors previously studied by Smith et al. [19] on the SA mediated endolysosomal escape of fluorescently labeled saporin and the saporin based anti-CD38 immunotoxin OKT10-SAP. CD38 is expressed by a variety of hematological malignancies and is an important potential target molecule for targeted toxin therapy. We have previously shown that the use of pulse shape analysis, in particular the measurement of fluorescent pulse width by flow cytometry, is capable of distinguishing between cells in which endolysosomal escape of a fluorescently labeled saporin or IT into the cytosol has occurred to cells in which the toxin remains confined to the endolysosomal compartment [20]. The fluorescent pulse width is representative of the duration for which a fluorescent signal is detected as a cell passes through the laser beam of the flow cytometer. The endolysosomal escape of the fluorescently labeled toxin into the cytoplasm results in a wider distribution throughout the cell, which is measured as an increase in the fluorescent pulse width by the flow cytometer. This technique was therefore used in this study to investigate the effect of six different pharmacological inhibitors with defined activities on the endolysosomal escape of fluorescently labeled saporin and OKT10-SAP.

## 2. Experimental Section

### 2.1. Materials

#### 2.1.1. Cell Lines

**Figure 1:**
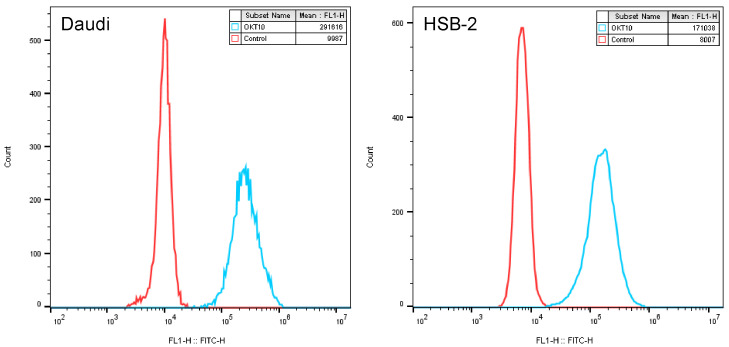
Expression of CD38 on Daudi and HSB-2 cells. Flow cytometry was used to assess the expression of CD38 on the surface of Daudi and HSB-2 cells by binding to the anti-CD38 antibody OKT10. Negative controls are presented for comparison.

The human Daudi Burkitt lymphoma cell line [21] and the T-cell acute lymphoblastic leukemia cell line HSB-2 [22] were obtained from the European Collection of Cell Cultures (ECACC, Porton Down, Salisbury, UK). Cell lines were authenticated using the Identifier Plus DNA profiling system (Applied Biosystems, Foster City, CA, USA). Working cell banks were produced and frozen in liquid nitrogen. Cultures were maintained in the logarithmic growth phase by regular passage in RPMI 1640 medium containing 10% fetal calf serum (FCS) and supplemented with 2 mM glutamine and 2 mM sodium pyruvate (referred to hereafter as R10) at 37 °C in 7% CO_2_ in a humidified environment for no longer than four weeks, following which a fresh vial of cells were resurrected from the working cell bank. The surface expression levels of CD38 for each cell line were measured by flow cytometry as previously described [16], and histograms are presented in Figure 1.

#### 2.1.2. Saponinum Album

Saponinum Album (SA), an extract of saponins from *Gypsophila paniculata* L. and Gypsophila arrostii Guss, was obtained as a commercial preparation from Merck (Darmstadt, Germany). SA contains a mixture of saponin species with the same aglycone core but varying carbohydrate side chains [23]. The structures of the most abundant of these, SA1641 and SA1657, have been described previously [13].

#### 2.1.3. Saporin

The SO6 isoform of saporin was extracted and purified from the seeds of *Saponaria officinalis* L. (Soapwort) (Chiltern Seeds, Ulverston, Cumbria, UK), as described elsewhere [7].

#### 2.1.4. Immunotoxin

The IgG1 murine monoclonal antibody OKT10 against human CD38 was produced from cultures of the OKT10 hybridoma cell line [16]. OKT10 was covalently coupled to the SO6 isoform of saporin using the heterobifunctional cross-linking reagent SPDP as described previously [24]. The antibody:toxin ratios of the resulting conjugate, termed OKT10-SAP, were previously determined to be, as a percentage of the total protein present: 1:1, ~55%, 1:2, ~10%, and ~15%, which could be either 1:3 or a 2:2 dimer. Alongside these conjugates, there was also determined to be ~10% free antibody and ~10% free saporin.

### 2.2. Methods

#### 2.2.1. Fluorescent Labeling of Saporin and OKT10-SAP

To detect the trafficking of internalized saporin and OKT10-SAP together with their proposed endolysosomal escape in the presence of SA, fluorescent conjugates were constructed with an Alexa Fluor 488 5-TFP (Life Technologies, Carlsbad, CA, USA) and termed SAP-AF and OKSAP-AF, respectively. This was achieved by adding 800 μL of 9.3 mg/mL saporin SO_6_ or 3.5 mg/mL OKT10-SAP to 100 μL carbonate buffer (1 M NaHCO_3_, pH 9.0) and 100 μL of Alexa Fluor 488 5-TFP (10 mg/mL in DMSO). Following stirring for 1 h at room temperature to effect conjugation, unconjugated fluorophore was removed by exhaustive dialysis for two hours at 4 °C against 2 L PBS followed by a further 2 L of PBS overnight at 4 °C. The concentrations of the resultant fluorescent conjugates were calculated using the Beer–Lambert law from their absorbance at 280 and 495 nm as measured on a Hitachi U1100 Spectrophotometer.

#### 2.2.2. Cell Culture

All experiments were conducted in phenolphthalein-free RPMI 1640 containing 10% FCS and supplemented with 2 mM glutamine and 2 mM sodium pyruvate.

#### 2.2.3. XTT Cytotoxicity Assay

Quadruplicate cultures of Daudi and HSB-2 cells (5 × 10^4^ cells per well) were seeded into 96 well plates in R10 and a dose-response titration with Saporin (1 × 10^−14^ M to 1 × 10^−5^ M) or OKT10-SAP (1 × 10^−16^ M to 1 × 10^−7^ M) was conducted in the presence or absence of 1 µg/mL of SA. Daudi cells were exposed continuously to 0.01 µM nocodazole, 0.005 µM bafilomycin A1, 25 µM EIPA, 100 µM chloroquine, 7.5 µM chlorpromazine, or 0.75 µM cytochalasin D, and HSB-2 cells to 0.01 µM nocodazole, 0.005 µM bafilomycin A1, 20 µM EIPA, 10 µM chloroquine, 7.5 µM chlorpromazine, or 0.75 µM cytochalasin D. Optimal inhibitor concentrations were previously determined by Smith et al. [19].

Plates were incubated for 48 h at 37 °C, 7% CO_2_. Cell viability was determined by a modified XTT assay as first described by Scudiero et al. [25]. Plates were read on a BMG Fluostar plate reader using a spectral scan from 300–650 nm. Results were expressed as a percentage of control cells cultured in the medium or SA alone and the 50% inhibitory concentration (EC50) was determined from the intercept with the 50% level on the Y axis of the dose–response curve. The fold increase was calculated by dividing the EC50 value for IT without SA by the EC50 value with SA. All experiments were repeated three times. The difference in fold increase between uninhibited control cells and cells treated with each inhibitor was analyzed by Mann–Whitney U-Test.

#### 2.2.4. Flow Cytometry

Daudi cells were incubated with 1 × 10^−6^ M SAP-AF or 5 × 10^−9^ M OKSAP-AF in R10 at 37 °C, 7% CO_2_ for 24 h. This was repeated with HSB-2 cells with 1 × 10^−6^ M SAP-AF or 5 × 10^−9^ M OKSAP-AF. Cells were washed and resuspended in R10 before being plated in 96 well plates at 1.25 × 105 cells per well with or without 1 µg/mL of SA in the presence or absence of an inhibitor with a final volume of 250 μL. Daudi cells were exposed to 0.01 µM nocodazole, 0.005 µM bafilomycin A1, 25 µM EIPA, 100 µM chloroquine, 7.5 µM chlorpromazine, or 0.75 µM cytochalasin D and HSB-2 cells to 0.01 µM nocodazole, 0.005 µM bafilomycin A1, 20 µM EIPA, 10 µM chloroquine, 7.5 µM chlorpromazine, or 0.75 µM cytochalasin D. Plates were incubated at 37 °C, 7% CO_2_, and cells removed from appropriate wells at 0, 6, 24, and 48 h after the addition of SA. Cells were washed, resuspended in 100 µL of RPMI-1640 in flow tubes, and analyzed at 10 µL/min on a Cytoflex flow cytometer (Beckman Coulter, Indianapolis, IN, USA) equipped with 488 nm 50 mW laser. Gates were set to exclude debris, and approximately 10,000 events were recorded per sample. Alexa Fluor 488 data was collected with a 525/40 nm bandpass filter with height (FITC-H), width (FITC-W), and area parameters recorded. Cytometer gain was calibrated daily against fluorescently labeled beads as per the manufacturer’s instructions. Data was recorded and analyzed using CytExpert software (Version 2.1.0.92, Beckman Coulter Life Sciences).

## 3. Results

### 3.1. Investigation of the Effects of Inhibitors of Endocytosis on the Endolysosomal Escape of SAP-AF and OKSAP-AF Measured by Pulse Shape Analysis

In order to investigate the effect of SA on the endolysosomal escape of saporin and OKT10-SAP into the cytosol, Daudi and HSB-2 cells were incubated with SAP-AF or OKSAP-AF for 24 h prior to treatment with 1 µg/mL of SA or mock treatment with R10 in the presence or absence of a range of inhibitors of endocytic mechanisms. The changes in FITC-W corresponding with endolysosomal escape were recorded immediately after the addition of SA and then after 6, 24, and 48 h.

**Figure 2:**
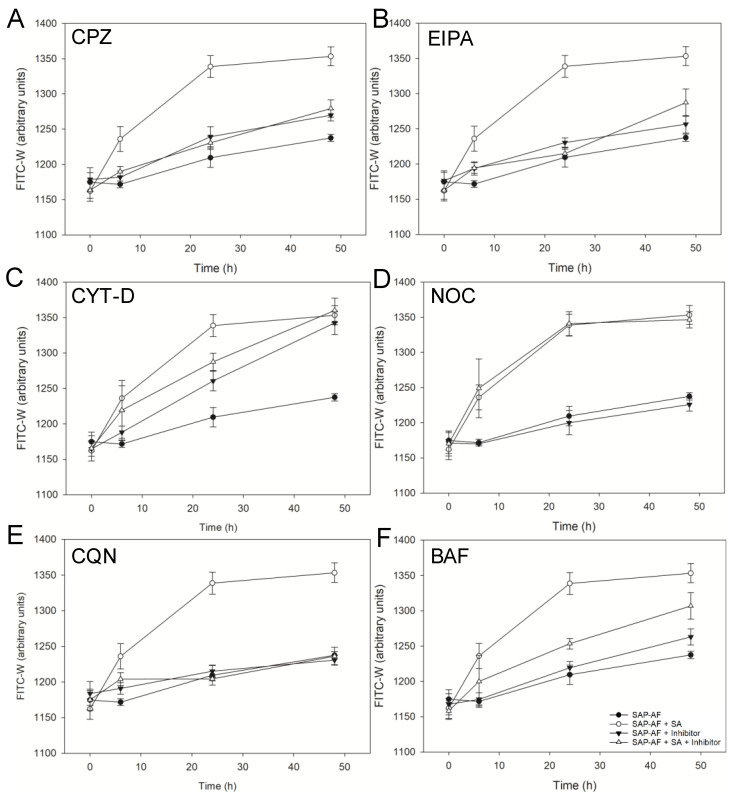
Investigation of the inhibition of SA mediated endolysosomal escape of SAP-AF in Daudi cells by pharmacological agents as measured by pulse width analysis. The effect of chlorpromazine (**A**), EIPA (**B**), cytochalasin D (**C**), nocadazole (**D**), chloroquine (**E**), and bafilomycin A1 (**F**) on the SA mediated endolysosomal escape of SAP-AF in Daudi cells. Charts show the changes in FITC-W over time in cells treated with 1 µg/mL of SA in the presence (△) and absence (○) of inhibitor. Untreated control cells are shown in each chart for comparison in the presence (▼) and absence (●) of inhibitor. Each datum point represents the calculated mean of three separate experiments, each performed in duplicate, and error bars one standard deviation either side of this mean.

**Figure 3:**
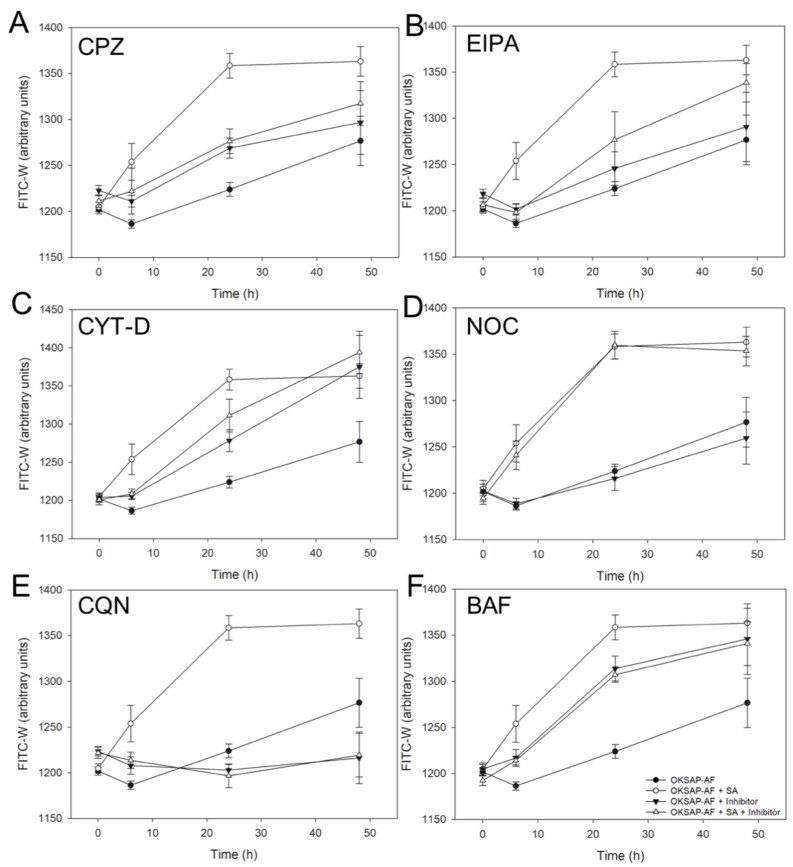
Investigation of the inhibition of SA mediated endolysosomal escape of OKSAP-AF in Daudi Cells by pharmacological agents as measured by pulse width analysis. The effect of chlorpromazine (**A**), EIPA (**B**), cytochalasin D (**C**), nocadazole (**D**), chloroquine (**E**), and bafilomycin A1 (**F**) on the SA mediated endolysosomal escape of OKSAP-AF in Daudi cells. Charts show the changes in FITC-W over time in cells treated with 1 µg/mL of SA in the presence (△) and absence (○) of inhibitor. Untreated control cells are shown in each chart for comparison in the presence (▼) and absence (●) of inhibitor. Each datum point represents the calculated mean of three experiments each performed in duplicate and error bars one standard deviation either side of this mean.

In Daudi cells loaded with either SAP-AF or OKSAP-AF an increase in median FITC-W was measured in the absence of SA after 24 h with a further increase by 48 h (Figure 2 and Figure 3). Treatment of both SAP-AF and OKSAP-AF positive Daudi cells with 1 µg/mL of SA resulted in a more rapid rise in median FITC-W with an increase over that seen in control cells being measured after 6 h. The increase in FITC-W continued until 24 h after initial exposure with no further increase observed between 24 and 48 h. In HSB-2 cells loaded with either SAP-AF or OKSAP-AF, an initial reduction in FITC-W was observed in untreated control cells after 6 h. This was followed by an increase in median FITC-W out to 48 h after the addition of SA (Figures S1 and S2). In cells containing OKSAP-AF, treatment with SA resulted in an increase in FITC-W compared to untreated control cells after 24 h (Figure S2). In cells containing SAP-AF no significant increase in median FITC-W is observed by 24 h but a significant difference was observed after 48 h (Supplementary Materials Figure S1).

To investigate the effect of a number of pharmacological inhibitors on the SA induced increase in endolysosomal escape of the toxin and immunotoxin, this increase in median FITC-W was used as a marker. Clathrin mediated endocytosis (CME) is the most extensively studied endocytic pathway, with well-defined molecular processes and cargo specificity; it is ubiquitous in eukaryotic cells [26]. Chlorpromazine is widely used as an inhibitor of CME and acts by causing the assembly of clathrin and AP2 complexes on endosomal membranes, thus depleting these critical components from the plasma membrane and preventing formation of clathrin coated pits and subsequent endocytosis [27]. Chlorpromazine completely prevented the increase in FITC-W caused by treatment with 1 µg/mL SA observed in Daudi cells loaded with SAP-AF or OKSAP-AF compared to untreated controls (Figure 2A). In HSB-2 cells, a similar reduction in the SA mediated increase in FITC-W was observed loaded with either OKSAP-AF or SAP-AF (Figures S1A and S2A).

The role of the non-selective endocytic pathway, macropinocytosis, in the augmentation of OKT10-SAP and saporin endolysosomal escape by SA was analyzed in Daudi and HSB-2 cells using EIPA, an analogue of amiloride. EIPA inhibits the Na+/H+ exchanger isoform 1 at the plasma membrane. This prevents the exchange of protons generated metabolically by actin polymerization and leads to sub-membranous acidification that inhibits the functions of the proteins Rac1 and Cdc42, resulting in the suppression of ruffle formation at the plasma membrane surface and subsequent inhibition of macropinocytosis [28,29]. In both Daudi and HSB-2 cells, EIPA completely abrogated the increase in FITC-W in SAP-AF loaded cells treated with 1 µg/mL of SA (Figure 2B and Figure S1B). For cells pre-incubated with OKSAP-AF, EIPA reduced the rate at which FITC-W increased in such a way that no increase was observed at 6 h. This is in comparison to control cells where the majority of the increase in FITC-W occurs within the first 6 h. However, by the 48-h time point, the median FITC-W of these EIPA treated cells reached the same level as that seen in control cells (Figure 3B and Figure S2B).

Actin polymerization is involved in a wide range of endocytic processes including macropinocytosis [28], phagocytosis [29], and clathrin independent endocytosis [29,30] and has been implicated in CME in some circumstances depending on the size of the cargo and the location of endocytosis [31,32]. The fungal alkaloid, cytochalasin D, which blocks the polymerization and elongation of actin microfilaments [33], was used to investigate the involvement of actin in the enhancement of OKT10-SAP and saporin endolysosomal escape by SA. In Daudi cells pre-loaded with SAP-AF or OKSAP-AF and treated with cytochalasin D in the absence of SA, there was a time dependent increase in FITC-W (Figure 2C and Figure 3C). The increase in FITC-W was likely due to cell swelling caused by cytochalasin D, which was measured as an increased forward scatter by flow cytometry (Figure S3). The presence of cytochalasin D prevented further increases in FITC-W due to the effect of SA on this cell line. In HSB-2 cells cytochalasin D did not increase the median FITC-W above that of control cells containing SAP-AF or OKSAP-AF in the absence of SA. In the presence of 1 µg/mL of SA, there was no further increase in FITC-W observed (Figures S1C and S2C).

Newly formed endocytic vesicles are transported away from the cortical region along microtubules. The inward trafficking and localization of endosomes as they mature from early to late endosomal compartments and finally to form the endolysosomal system requires their active motility along microtubules [34]. Treatment with the microtubule disrupting drug nocodazole has been shown to prevent transport to the endolysosomal compartment [30]. Nocadazole was used here with the aim of preventing the trafficking of SA to an acidic endolysosomal compartment where endosomal escape has been reported to occur [13]. In Daudi cells, nocadazole had no significant effect on the increased FITC-W observed with SA in cells loaded with either SAP-AF or OKSAP-AF (Figure 2D and Figure 3D). In HSB-2 cells a significant change in the increase in FITC-W was only seen in cells pre-incubated with SAP-AF and treated with 1 µg/mL of SA (Figures S1D and S2D).

To investigate the role of a low endolysosomal pH on the augmentation of OKT10-SAP and saporin cytotoxicity by SA, two pharmacological inhibitors of endolysosomal acidification were used. Daudi cells were incubated with either bafilomycin A1, a specific inhibitor of the vacuolar H+ ATPase [32], or with the lysosomotropic drug chloroquine, which diffuses into the endolysosomal compartment, where it becomes protonated thereby directly increasing endolysosomal pH [31]. In the presence of these inhibitors, the effect of SA on the endolysosomal escape of OKSAP-AF and SAP-AF was evaluated by flow cytometry.

Treatment of OKSAP-AF loaded Daudi cells with bafilomycin A1 in the absence of SA resulted in an increase in FITC-W (*p* = 0.015, two-way ANOVA) (Figure 3F). Confocal microscopy of these cells suggested that bafilomycin A1 might cause OKSAP-AF loaded vesicles to become dispersed widely throughout the cytosol, in comparison to their more localized, peri-nuclear distribution observed in control cells (Figure S4). In these Daudi cells, the presence of bafilomycin A1 prevented any further increase in FITC-W from SA 1 µg/mL compared to untreated control cells, and there was also a significant reduction in FITC-W compared to cells treated with SA alone. This suggests that bafilomycin A1 prevents endolysosomal escape of OKSAP-AF at this concentration of SA. Bafilomycin A1 did not significantly increase FITC-W in the absence of SA in Daudi cells loaded with SAP-AF (Figure 2F). The inhibitor significantly reduced the increase due to 1 µg/mL SA. Bafilomycin A1 alone did not increase FITC-W in HSB-2 cells pre-loaded with SAP-AF or OKSAP-AF. In cells pre-incubated with SAP-AF, bafilomycin A1 did not significantly reduce the increase in FITC-W caused by treatment with 1 µg/mL of SA. In contrast, in cells loaded with OKSAP-AF, bafilomycin A1 significantly reduced the increase in FITC-W caused by this concentration of SA. In both Daudi (Figure 2E and Figure 3E) and HSB-2 cells (Figures S1E and S2E), chloroquine completely abrogated the increase in FITC-W seen for both SAP-AF and OKSAP-AF with 1 µg/mL SA.

### 3.2. Effects of Small Molecule Pharmacological Agents on the Augmentation of OKT10-SAP and Saporin Cytotoxicity by SA

The effect of each of these inhibitors on the augmentation of OKT10-SAP and saporin cytotoxicity by 1 µg/mL of SA was then investigated in Daudi and HSB-2 cells in order that they could be compared to the effects of the inhibitors on the endolysosomal escape of the toxin.

**Figure 4:**
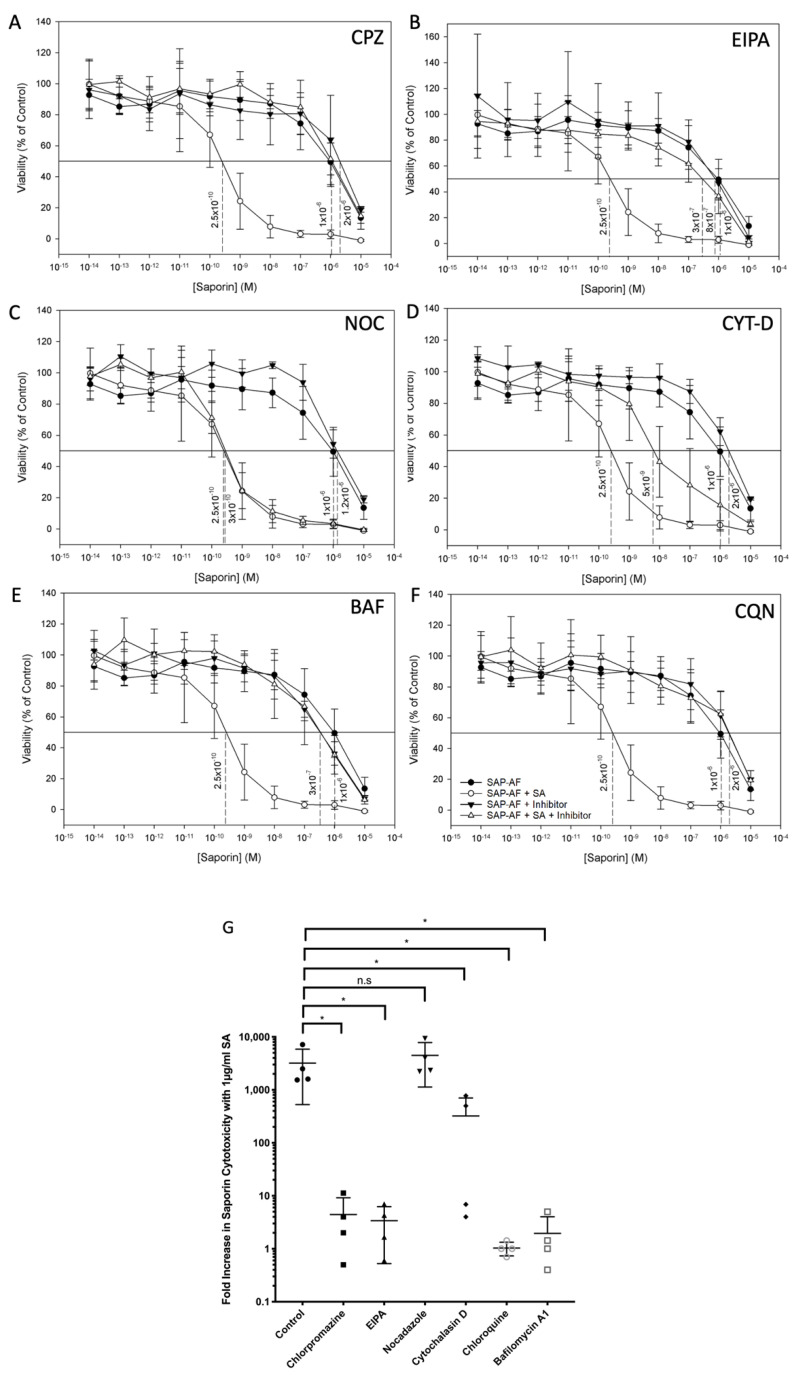
Inhibition of SA-mediated augmentation of saporin cytotoxicity in Daudi Cells by the various pharmacological agents studied. Effect of chlorpromazine (**A**), EIPA (**B**), nocodazole (**C**), cytochalasin D (**D**), bafilomycin A1 (**E**), and chloroquine (**F**) on the cytotoxicity of saporin and saporin used in combination with 1 µg/mL of SA. (**A**–**F**): Dose–response curves determined by XTT assay for saporin on Daudi lymphoma cells with each agent in the absence (▼) and presence (△) of SA. In each chart, the data for saporin without each agent in the absence (●) and presence (○) of SA are also presented for comparison. Each datum point represents the calculated mean of four experiments each performed in quadruplicate cell cultures and the error bars one standard deviation either side of this mean. The EC50 obtained from each curve is shown against the perpendicular dotted line. (**G**): Fold increases in saporin cytotoxicity with 1µg/mL SA in control (●), chlorpromazine (■), EIPA (▲), nocodazole (▼), cytochalasin D (◆), chloroquine (○), and bafilomycin A1 (□). Dots represent fold increase for individual experiments with the lines showing the mean and one standard deviation either side of this mean. Augmentation was significantly abrogated by chlorpromazine (*p* = 0.0286 *), EIPA (*p* = 0.0286 *), cytochalasin D (*p* = 0.0286 *), chloroquine (*p* = 0.0286 *), and bafilomycin A1 (*p* = 0.0286 *) as determined by Mann–Whitney U-Test. CPZ: chlorpromazine; CQN: chloroquine; BAF: bafilomycin A1; CYT-D: cytochalasin D; NOC: nocadazole.

**Figure 5:**
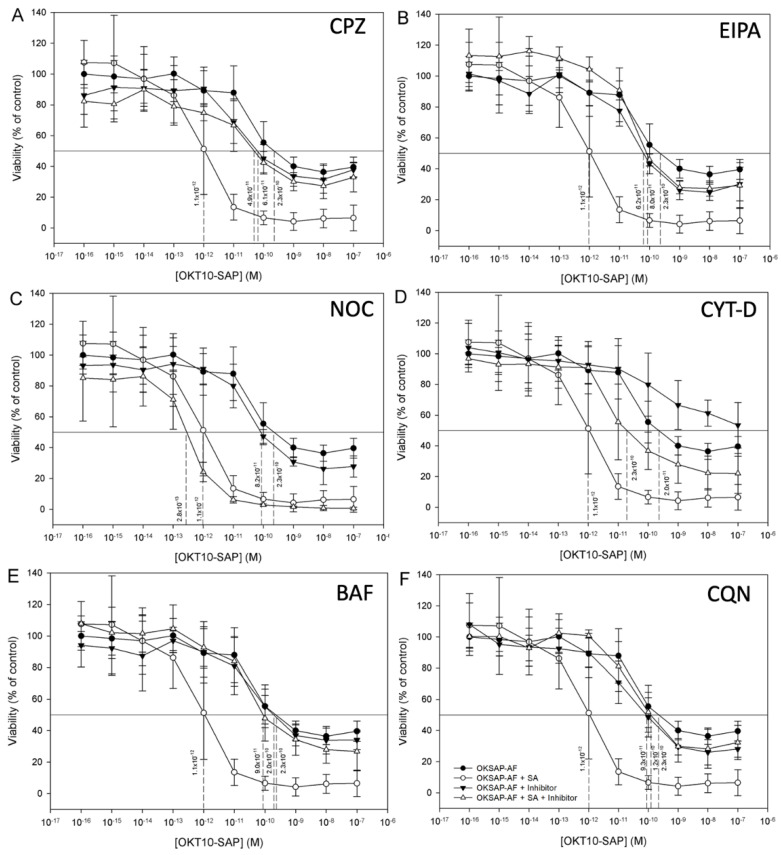

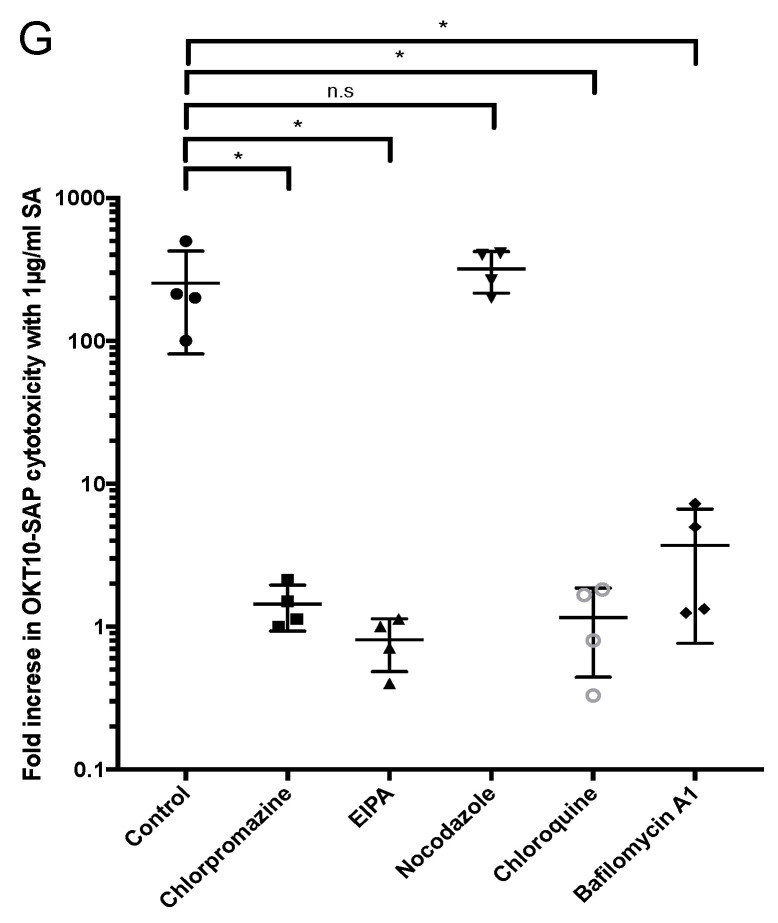
Inhibition of SA-Mediated augmentation of OKT10-SAP cytotoxicity in Daudi cells by the various pharmacological agents studied. Effect of chlorpromazine (**A**), EIPA (**B**), nocodazole (**C**), cytochalasin D (**D**), bafilomycin A1 (**E**), and chloroquine (**F**) on the cytotoxicity of OKT10-SAP and OKT10-SAP used in combination with 1 µg/mL of SA. (**A**–**F**): Dose–response curves determined by XTT assay for OKT10-SAP on Daudi lymphoma cells with each agent in the absence (▼) and presence (△) of SA. In each chart the data for OKT10-SAP without inhibitor in the absence (●) and presence (○) of SA is also presented for comparison. Each datum point represents the calculated mean of four separate experiments each performed in quadruplicate cell cultures. Error bars represent one standard deviation either side of this mean. The EC50 obtained from each curve is shown against the perpendicular dotted line. (**G**): Fold increases in OKT10-SAP cytotoxicity with 1µg/mL SA in control (●), chlorpromazine (■), EIPA (▲), nocodazole (▼), chloroquine (○), and bafilomycin A1 (◆). Dots represent fold increase in individual experiments with the lines showing the mean and one standard deviation either side of this mean. Augmentation was significantly abrogated by chlorpromazine (*p* = 0.0286 *), EIPA (*p* = 0.0286 *), chloroquine (*p* = 0.0286 *), and bafilomycin A1 (*p* = 0.0286 *) as determined by Mann–Whitney U-Test. CPZ: chlorpromazine; CQN: chloroquine; BAF: bafilomycin A1; CYT-D: cytochalasin D; NOC: nocadazole.

The effect of the CME inhibitor chlorpromazine on the augmentation of OKT10-SAP and saporin cytotoxicity by SA in Daudi and HSB-2 cells was investigated by XTT assay. Treatment of both Daudi and HSB-2 cells with chlorpromazine did not affect their sensitivity to the cytotoxicity of OKT10-SAP or saporin, suggesting that the cytotoxic activity of OKT10-SAP or saporin is not clathrin dependent. In contrast, the augmentation of both OKT10-SAP and saporin cytotoxicity by SA was almost completely abrogated in both Daudi cells (Figure 4A and Figure 5A) and HSB-2 cells (Figures S5A and S6A).

Incubation of Daudi and HSB2 cells with the macropinocytosis inhibitor EIPA did not affect the toxicity of OKT10-SAP or saporin alone. However, EIPA completely abrogated augmentation of OKT10-SAP (Figure 5B and Figure S6B) and almost completely abrogated augmentation of saporin by 1 µg/mL SA (Figure 4B and Figure S5B).

In Daudi cells, the actin inhibitor cytochalasin D significantly reduced the toxicity of OKT10-SAP alone (*p* = 0.0038, two-way ANOVA) and when used in combination with SA (*p* = 0.0002, two-way ANOVA) (Figure 5D). A similar reduction in the cytotoxicity of OKT10-SAP alone was observed in HSB-2 cells (*p* < 0.0001, two-way ANOVA) and with IT in combination with 1 µg/mL of SA (*p* < 0.0001, two-way ANOVA). It was not possible to calculate a mean fold increase for the effect of SA on OKT10-SAP EC50 in the presence of cytochalasin D in Daudi cells, as the EC50 was not reached over the concentration range tested (1 × 10^−16^ M–1 × 10^−7^ M) (Figure 5D). In comparison to its effect on OKT10-SAP, cytochalasin D did not reduce the cytotoxicity of the native toxin in the absence of SA. In the presence of SA, treatment with cytochalasin D partially abrogated the augmentation of saporin cytotoxicity in Daudi cells (Figure 4D) and almost completely abrogated augmentation in HSB-2 cells (Figure S5D).

The microtubule inhibitor nocadazole had no effect on the cytotoxicity of OKT10-SAP or saporin either alone or in combination with SA in either Daudi cells (Figure 4C and Figure 5C) or HSB-2 cells (Figures S5 and S6).

The role of a low endolysosomal pH on the augmentation of OKT10-SAP and saporin cytotoxicity by 1 µg/mL SA was evaluated by XTT assay in the presence of the inhibitors bafilomycin A1 and chloroquine. Neither of these pharmacological agents affected the cytotoxicity of either OKT10-SAP or saporin alone in Daudi or HSB-2 cells. However, both agents greatly reduced the SA-mediated augmentation of OKT10-SAP in Daudi (Figure 5E,F) and HSB-2 cells (Figure S6E,F). In Daudi cells, the augmentation of saporin cytotoxicity by SA was almost completely abrogated by both agents (Figure 4E,F). In HSB-2 cells, complete abrogation the augmentation of saporin cytotoxicity was seen with chloroquine (Figure S5E), whilst a partial abrogation was seen with bafilomycin A1 (Figure S5F).

### 3.3. Comparison of the Effect of Pharmacological Agents on the SA-Mediated Augmentation of Cytotoxicity and Its Correlation with the Observed Endolysosomal Escape of Saporin and OKT10-SAP

**Figure 6:**
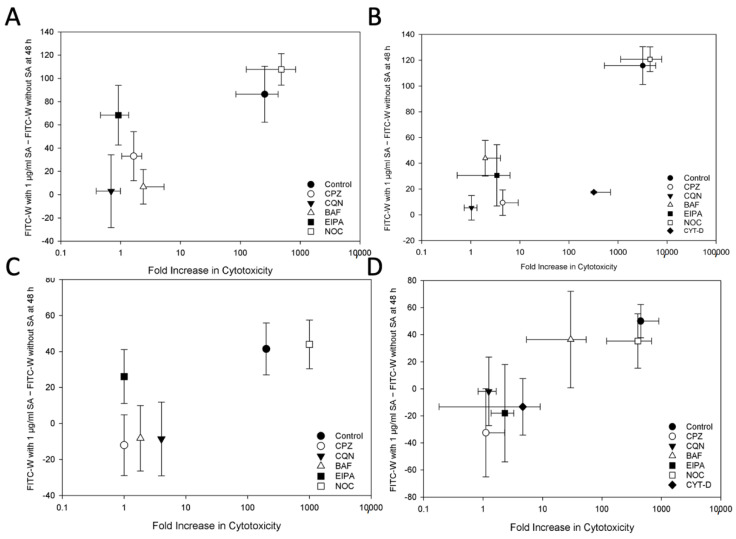
Correlation between the effect of pharmacological agents on the SA-Mediated augmentation of saporin and OKT10-SAP cytotoxicity and on the SA-mediated Increase in endolysosomal escape of SAP-AF and OKSAP-AF. Scatter graphs comparing the SA-mediated fold increase in cytoxicity versus the SA-mediated difference in FITC-W after 48 h of exposure to 1 µg/mL. (**A**,**B**) show the data for Daudi cells with OKT10-SAP/OKSAP-AF and saporin/SAP-AF, respectively, with (**C**,**D**) showing the data for HSB-2 cells with OKT10-SAP/OKSAP-AF and saporin/SAP-AF, respectively. Each data point shows the mean fold increase in cytotoxicity from four independent XTT assays and the mean difference in FITC-W from three independent flow cytometry studies with each inhibitor and untreated controls included for comparison. These experiments were not paired. Error bars represent the standard deviation from the mean. No error bars are presented for the fold increase in cytotoxicity for OKT10-SAP on HSB-2 cells (**C**) as an EC50 was achieved in only some of the experiments performed. CPZ: chlorpromazine; CQN: chloroquine; BAF: bafilomycin A1; CYT-D: cytochalasin D; NOC: nocadazole.

A comparison was made between the effects of each of the pharmacological agents used in this study on the augmentation of saporin and OKT10-SAP cytotoxicity by SA and their effect on the SA-mediated increase in FITC-W as a marker for endolysosomal escape. The accompaniment of an inhibition of cytotoxicity augmentation by a corresponding inhibition of endolysosomal escape would provide further evidence that enhancement of endolysosomal escape is the mechanism at least partially responsible for the SA-mediated augmentation of saporin cytotoxicity. To compare the changes in these variables, the fold increase in EC50 in cells treated with saporin or OKT10-SAP in the presence of 1 µg/mL of SA was used as a marker for the augmentation of cytotoxicity. As a marker for the effect of SA on endolysosomal escape, the difference in the FITC-W between cells loaded with SAP-AF or OKSAP-AF and treated with SA for 48 h and the FITC-W of cells mock treated with R10 for the same time period, in the presence of each inhibitor, was calculated. From these comparisons, a clear trend can be observed. Pharmacological agents that abrogated the SA-mediated augmentation of saporin and OKT10-SAP cytotoxicity also reduced the SA-mediated increase in FITC-W corresponding with the increased endolysosomal escape of SAP-AF and OKSAP-AF (Figure 6). Similarly, for both cell lines, nocadazole, which had no effect on the augmentation of the cytotoxicity of saporin and OKT10-SAP also had no effect on their SA-mediated endolysosomal escape.

Several exceptions to this trend were observed. In both Daudi and HSB-2 cells, EIPA abrogated the SA-mediated augmentation of OKT10-SAP cytotoxicity but did not significantly reduce the endolysosomal escape of OKSAP-AF after 48 h (Figure 6A,C). EIPA reduced the SA-mediated increase in FITC-W for OKT10-SAP at earlier time points, suggesting that this agent reduced the rate of endolysosomal escape of the IT in these cells. This would delay the cytotoxic effect of the toxin, and this may therefore explain the discrepancy between the effect of EIPA on endolysosomal escape and on the augmentation of cytotoxicity. In HSB-2 cells, bafilomycin A1 only partially though still significantly abrogated the cytotoxicity of saporin but failed to reduce the SA-mediated increase in FITC-W for SAP-AF (Figure 6D). Another exception to the observed trend was the effect of cytochalasin D on saporin in Daudi cells (Figure 6B). This agent slightly reduced the augmentation of saporin cytotoxicity by SA, but appeared to almost completely abrogate the SA-mediated increase in FITC-W. Cytochalasin D also increased the FITC-W of cells untreated with SA, and this would have reduced the difference in FITC-W between SA treated and untreated cells.

## 4. Discussion

The aim of this work was to determine the effects of small molecule pharmacological agents with defined activities against endocytic and other cellular processes on the ability of triterpenoid saponins to promote the endolysosomal release of saporin to the cytosol of target lymphoma cells. One proposed hypothesis for the mechanism by which saponins augment the cytotoxicity of saporin and saporin based ITs requires that both saporin and saponin are internalized by cells and trafficked together through the same sequential endosomal compartments to the endolysosome. Here, saponin promotes the endolysosomal escape of saporin into the cytosol by a currently unknown mechanism that may involve a pH dependent non-covalent association between saponin and the toxin. This study made use of a number of pharmacological agents to probe the roles of different endocytic processes and endosomal acidification on the SA mediated enhancement of endolysosomal escape of saporin to the cytosol and to compare the results of these experiments with the effect that each pharmacological agent has on the SA-mediated augmentation of toxin cytotoxicity.

Treatment with chlorpromazine both completely prevented the endolysosomal escape of SAP-AF and OKSAP-AF in cells exposed to 1 µg/mL SA and near completely abrogated the SA-mediated augmentation of saporin and OKT10-SAP cytotoxicity. This implies that the abrogatory effect of chlorpromazine on SA-mediated augmentation of toxin cytotoxicity is directly linked to inhibition of the endolysosomal escape of the toxin by this agent. A firm conclusion about the role of CME in augmentation is, however, confounded by the results that were obtained when inhibiting macropinocytosis with EIPA. EIPA significantly abrogated the increase in FITC-W in cells loaded with SAP-AF or OKSAP-AF and treated with 1 µg/mL of SA. Similarly, treatment of cells with EIPA abrogated the SA-mediated augmentation of saporin and OKT10-SAP cytotoxicity, but did not reduce the cytotoxicity of the toxin alone. These results suggest that the effect of EIPA on the augmentation of cytotoxicity by SA is also due, at least in part, to its effect on the SA mediated endolysosomal escape of the toxin. It is entirely possible that both CME and macropinocytosis are both involved in the internalization of SA. However, the near complete abrogation of both the SA mediated increase in toxin and IT endolysosomal escape and SA mediated augmentation of cytotoxicity by both chlorpromazine and EIPA would make this seem unlikely. This makes it difficult to draw any firm conclusion as to whether one or both processes are involved in augmentation. One possible explanation for these results is that either or both of chlorpromazine and EIPA are exerting an off-target inhibition of the other endocytic pathway or that both inhibitors may be affecting an unidentified, separate process that is involved as an augmentation mechanism.

Whilst both chlorpromazine and EIPA are often used as specific inhibitors of CME [27,35] and macropinocytosis [36,37], respectively, they are both known to exert other cellular effects [37]. Chlorpromazine is amphipathic and incorporates into lipid bilayers resulting in increased lipid fluidity of the plasma membrane [38,39]. Changes to lipid fluidity have been shown to affect fluid phase endocytosis and exocytic processes [40]. Chlorpromazine is also a known inhibitor of calmodulin [35] and is known to inhibit phospholipase C (PLC) [41]. PLC is involved in activation of actin modifying proteins [42] and is necessary for the actin cytoskeletal reorganization in membrane ruffling and macropinocytosis in fibroblasts [43]. It is also of interest that chlorpromazine has been shown to inhibit exogenous cholesterol esterification and sphingomyelinase activity [44] together with the effect of depleting cells of un-esterified cholesterol by increasing the net transfer of cholesterol from cells to the serum [45]. This is particularly relevant, as the augmentation of saporin based IT’s cytotoxicity by SA has recently been demonstrated to be dependent on plasma membrane cholesterol content [46]. EIPA cannot be considered to specifically or directly inhibit macropinocytosis, as it acts by inhibiting the Na+/H+ exchanger (NHE1) at the plasma membrane causing sub-membranous acidification, leading to the inhibition of ruffle formation in the cell membrane and macropinocytosis [47]. Inhibition of the NHE1 by EIPA has been shown to affect the actin cytoskeleton [48], which may in turn lead to a non-specific inhibition of endocytosis.

In Daudi cells, the effect of the actin disrupting agent cytochalasin-D on the SA induced increase FITC-W was masked by its effect on the volume of control cells. However, in HSB-2 cells treatment with cytochalasin D resulted in the complete abrogation of the increase in FITC-W observed in cells loaded with either SAP-AF or OKSAP-AF and treated with 1 µg/mL SA. These results concur with the effect of cytochalasin D on the SA-mediated augmentation of cytotoxicity, which was observed to vary between cell lines. In Daudi cells, only a partial abrogation of the augmentation of both saporin and OKT10-SAP cytotoxicity was achieved with cytochalasin D. By comparison, in HSB-2 cells, complete inhibition of augmentation of cytotoxicity was observed with cytochalasin-D. This suggests that actin polymerization is involved in the SA induced endolysosomal escape of these conjugates and the resulting augmentation of their cytotoxicity, most likely through inhibition of the internalization and trafficking of SA to the endolysosomal compartment. However, this does not allow for an analysis that would provide discrimination between pathways. This is further confounded by the fact that actin is also known to be involved in the formation of clathrin coated pits and treatment of cells with actin disrupting agents can also reduce the efficiency of CME and has been shown to inhibit the uptake of transferrin by this endocytic pathway [49]. Furthermore, the effect of cytochalasin D on CME has been shown be variable between cell lines suggesting that the role of actin in CME is not obligatory [50].

In both Daudi and HSB-2 cells the microtubule inhibitor nocadazole did not abrogate the SA-mediated augmentation of OKT10-SAP or saporin cytotoxicity or the endolysosomal escape of SAP-AF or OKSAP-AF. These results suggest that microtubule-dependent trafficking is not required for saporin/immunotoxin cytotoxicity or for SA-mediated cytotoxicity. It has been previously reported that nocadazole inhibits the trafficking of cargo between the early and late endosome compartments, but not the transport of cargo from the plasma membrane to the early endosome [51]. Additionally, Weng et al. reported that the preincubation of ECV-304 cells with nocadazole prior to their exposure to an Alexa Fluor labeled, histidine tagged saporin resulted in its accumulation in larger vesicles than in untreated cells [52]. However, these workers did not conduct co-localization studies, so it is not possible to say definitively whether these vesicles represented early endosomes, but if so, then this might suggest that nocadazole prevents the transfer of saporin between the early and late endosomal compartments. The lack of any inhibitory effect of nocadazole on the augmentation of saporin or OKT10-SAP by SA suggests that the trafficking of the toxin and SA to the late endosome or lysosome is not required for augmentation. This finding indicates that SA-mediated escape of saporin may occur from the early endosomal compartment. This would run contrary to the hypothesis that trafficking of the saporin and SA to the more acidic endolysosomal compartment is a prerequisite for SA-mediated cytotoxicity augmentation.

The role of endosomal acidification on the SA mediated increase in toxin endolysosomal escape was investigated with two pharmacological agents, chloroquine and bafilomycin A1. Of these, chloroquine completely inhibited the SA-mediated increase in FITC-W with either SAP-AF or OKSAP-AF and significantly abrogated the augmentation of saporin and OKT10-SAP cytotoxicity by SA. The effect of bafilomycin A1 was less clear-cut. In cytotoxicity experiments, bafilomycin A1 significantly abrogated the augmentation of saporin and OKT10-SAP cytotoxicity by SA in Daudi cells. However, in Daudi cells incubated with OKSAP-AF, treatment with bafilomycin A1 increased FITC-W in the absence of SA. Confocal microscopy studies suggested that this may be due to a dispersal of OKSAP-AF containing vesicles throughout the cell in the presence of bafilomycin A1 and highlights the need for fluorescent microscopy to be used alongside pulse shape analysis flow cytometry to exclude other causes of FITC-W changes wherever this is possible. In addition, the inability of bafilomycin A1 to significantly reduce the SA-mediated increase in FITC-W in HSB-2 cells containing SAP-AF did not correspond with the effect of the inhibitor in XTT cytotoxicity assays. In contrast, bafilomycin A1 almost completely abrogated the augmentation of saporin cytotoxicity with SA used at 1 µg/mL. The reason for this discrepancy is uncertain and warrants further investigation. However, despite this isolated result, the overall conclusion from these studies is that prevention of endosomal acidification inhibits the SA-mediated increase in FITC-W and therefore endolysosomal escape of toxin to the cytosol with a resultant augmentation of cytotoxicity. However, it should be noted that this does not inform us as to whether it is a direct, pH dependent effect on saporin or saponin that is the driving mechanism behind augmentation. The pH of endosomal contents and the pH differential between different vesicular compartments is important for a wide range of processes including enzyme activity and co-ordination of recycling between endosomes and the extracellular milieu [53]. Disruption of the normal endosomal pH by these inhibitors could therefore affect a number of different processes that might themselves play an important role in SA-mediated augmentation of saporin cytotoxicity. Both bafilomycin A1 and chloroquine inhibit autophagy via their effects on lysosomal pH [54,55], and this may be relevant considering the potential for saporin to cause cell death by multiple death pathways [56]. Additionally, Bafilomycin A1 has previously been shown to inhibit the transfer of endosomal contents from the early endosome to endosomal carrier vesicles, thus preventing their trafficking to the late endosomal compartment [51]. A similar effect has been demonstrated with chloroquine, which blocks the transfer of endocytosed material from early to late endosomal compartments resulting in their accumulation in the early endosome [57,58]. Therefore, these inhibitors of endosomal acidification might prevent the trafficking of saporin and/or saponin to later endosomal compartments, thereby preventing saponin achieving a sufficiently high concentration in the endosomal lumen to permeabilize the endosomal membrane. A summary of the effects of these inhibitors has been included in Table 1.

Comparison of the effect of each pharmacological agent and the correlation between toxin augmentation and escape indicates that increasing endolysosomal escape of the toxin is at least one of the contributory mechanisms by which SA augments saporin cytotoxicity, though it should be kept in mind that there may be additional mechanisms operating in parallel that have not been revealed by this study. Further work investigating these mechanisms should include genetic knockdown studies of various key molecular components of endocytic processes. The genetic knockdown approach would overcome the issue of specificity and other cellular effects exerted by small molecule pharmacological agents and provide a clearer picture of the roles that the various endocytic pathways play in the augmentation of saporin cytotoxicity by saponins.

## Figures and Tables

**Table 1:** The abrogatory effects of small molecule inhibitors on the endolysosomal escape of Saporin and OKT10-SAP in Daudi and HSB-2 cells. Strong abrogation (++), partial abrogation (+), no abrogation (−).

		Daudi		HSB-2	
		Abrogation of SA Mediated Augmentation of Saporin and OKT1-SAP			
Inhibitor	Inhibitor of	Endolysosomal Escape	Cytotoxicity	Endolysosomal Escape	Cytotoxicity
Chlorpromazine	CME	++	++	++	++
EIPA	Macropinocytosis	++	++	++	++
Cytochalasin-D	Actin polymerisation	-	+	++	++
Nocadazole	Microtubules	-	-	-	-
Chloroquine	Endosomal acidification	++	++	++	++
Bafilomycin-A1	Endosomal acidification	+	++	+	++

## Data Availability

The data presented in this study are available on request from the corresponding author.

## Supplementary Materials

The following are available online at https://www.mdpi.com/2227-9059/9/3/300/s1: Figure S1: Investigation of the Inhibition of SA Mediated Endolysosomal Escape of SAP-AF in HSB-2 Cells by Pharmacological Agents as Measured by Pulse Width Analysis, Figure S2: Investigation of the Inhibition of SA Mediated Endolysosomal Escape of OKSAP-AF in HSB-2 Cells by Pharmacological Agents as Measured by Pulse Width Analysis, Figure S3: Effect of Cytochalasin D on Daudi Cell Morphology, Figure S4: Confocal Microscopy Showing the Effect of Bafilomycin A1 on Daudi Cells Containing OKSAP-AF, Figure S5: Inhibition of SA-Mediated Augmentation of Saporin Cytotoxicity in HSB-2 Cells by the Various Pharmacological Agents Studied, Figure S6: Inhibition of SA-Mediated Augmentation of OKT10-SAP Cytotoxicity in HSB-2 Cells by the Various Pharmacological Agents Studied.

## Abbreviations

BAF	Bafilomycin A1
CME	Clathrin mediated endocytosis
CQN	Chloroquine
CPZ	Chlorpromazine
CYT-D	Cytochalasin D
EIPA	Ethylisopropylamiloride
FCS	Foetal calf serum
FITC	Fluorescein isothiocyanate
FITC-H	Pulse Height of the signal recorded by a 525/40 nm bandpass filter
FITC-W	Pulse Width of the signal recorded by a 525/40 nm bandpass filter
IT	Immunotoxin
NOC	Nocadazole
OKSAP-AF	Alexa Fluor 488 conjugated OKT10-SAP
PBS	Phosphate buffered saline
R10	RMPI-1640 medium supplemented with 10% FCS and 2 mM glutamine and 2 mM sodium pyruvate
RIP	Ribosome inactivating protein
RPMI	Roswell Park Memorial Institute Medium
SA	*Saponinum album*
SAP-AF	Alexa Fluor 488 conjugated Saponinum album
XTT	2,3-Bis-(2-Methoxy-4-Nitro-5-Sulfophenyl)-2H-Tetrazolium-5- Carboxanilide

## References

[1] Kreitman R.J. (2006). Immunotoxins for targeted cancer therapy. AAPS J..

[2] Stone M.J., Sausville E.A., Fay J.W., Headlee D., Collins R.H., Figg W.D., Stetler-Stevenson M., Jain V., Jaffe E.S., Solomon D. (1996). A phase I study of bolus versus continuous infusion of the anti-CD19 immunotoxin, IgG-HD37-dgA, in patients with B-cell lymphoma. Blood.

[3] Kreitman R.J., Stetler-Stevenson M., Margulies I., Noel P., FitzGerald D.J., Wilson W.H., Pastan I. (2009). Phase II trial of recombinant immunotoxin RFB4(dsFv)-PE38 (BL22) in patients with hairy cell leukemia. J. Clin. Oncol..

[4] Avarbock A.B., Loren A.W., Park J.Y., Junkins-Hopkins J.M., Choi J., Litzky L.A., Rook A.H. (2008). Lethal vascular leak syndrome after denileukin diftitox administration to a patient with cutaneous gamma/delta T-cell lymphoma and occult cirrhosis. Am. J. Hematol..

[5] Olsen E., Duvic M., Frankel A., Kim Y., Martin A., Vonderheid E., Jegasothy B., Wood G., Gordon M., Heald P. (2001). Pivotal Phase III Trial of two dose levels of denileukin diftitox for the treatment of cutaneous t-cell lymphoma. J. Clin. Oncol..

[6] Vallera D.A., Kreitman R.J. (2018). Immunotoxins targeting B cell malignancy—Progress and problems with immunogenicity. Biomedicines.

[7] Stirpe F., Gasperi-Campani A., Barbieri L., Falasca A., Abbondanza A., Stevens W.A. (1983). Ribosome-inactivating proteins from the seeds of *Saponaria officinalis* L. (soapwort), of *Agrostemma githago* L. (corn cockle) and of *Asparagus officinalis* L. (asparagus), and from the latex of *Hura crepitans* L. (sandbox tree). Biochem. J..

[8] Santanché S., Bellelli A., Brunori M. (1997). The Unusual stability of Saporin, a candidate for the synthesis of immunotoxins. Biochem. Biophys. Res. Commun..

[9] Bolognesi A., Tazzari P.L., Tassi C., Gromo G., Gobbi M., Stirpe F. (2008). A comparison of anti-lymphocyte immunotoxins containing different ribosome-inactivating proteins and antibodies. Clin. Exp. Immunol..

[10] Maras B., Ippoliti R., De Luca E., Lendaro E., Bellelli A., Barra D., Bossa F., Brunori M. (1990). The amino acid sequence of a ribosome-inactivating protein from *Saponaria officinalis* seeds. Biochem. Int..

[11] Savino C., Federici L., Ippoliti R., Lendaro E., Tsernoglou D. (2000). The crystal structure of Saporin SO6 from *Saponaria officinalis* and its interaction with the ribosome. FEBS Lett..

[12] Montanaro L., Sperti S., Mattioli A., Testoni G., Stirpe F. (1975). Inhibition by ricin of protein synthesis in vitro. Inhibition of the binding of elongation factor 2 and of adenosine diphosphate-ribosylated elongation factor 2 to ribosomes. Biochem. J..

[13] Weng A., Thakur M., von Mallinckrodt B., Beceren-Braun F., Gilabert-Oriol R., Wiesner B., Eichhorst J., Böttger S., Melzig M.F., Fuchs H. (2012). Saponins modulate the intracellular trafficking of protein toxins. J. Control. Release.

[14] Thakur M., Weng A., Pieper A., Mergel K., Von Mallinckrodt B., Gilabert-Oriol R., Görick C., Wiesner B., Eichhorst J., Melzig M.F. (2013). Macromolecular interactions of triterpenoids and targeted toxins: Role of saponins charge. Int. J. Biol. Macromol..

[15] Heisler I., Sutherland M., Bachran C., Hebestreit P., Schnitger A., Melzig M.F., Fuchs H. (2005). Combined application of saponin and chimeric toxins drastically enhances the targeted cytotoxicity on tumor cells. J. Control. Release.

[16] Holmes S.E., Bachran C., Fuchs H., Weng A., Melzig M.F., Flavell S.U., Flavell D.J. (2014). Triterpenoid saponin augmentation of saporin-based immunotoxin cytotoxicity for human leukaemia and lymphoma cells is partially immunospecific and target molecule dependent. Immunopharmacol. Immunotoxicol..

[17] Weng A., Thakur M., Beceren-Braun F., Bachran D., Bachran C., Riese S.B., Jenett-Siems K., Gilabert-Oriol R., Melzig M.F., Fuchs H. (2012). The toxin component of targeted anti-tumor toxins determines their efficacy increase by saponins. Mol. Oncol..

[18] Bachran D., Schneider S., Bachran C., Weng A., Melzig M.F., Fuchs H. (2011). The endocytic uptake pathways of targeted toxins are influenced by synergistically acting *Gypsophila* Saponins. Mol. Pharm..

[19] Smith W.S., Johnston D.A., Holmes S.E., Wensley H.J., Flavell S.U., Flavell D.J. (2019). Augmentation of saporin-based immunotoxins for human leukaemia and lymphoma cells by triterpenoid saponins: The Modifying effects of small molecule pharmacological agents. Toxins.

[20] Wensley H.J., Johnston D.A., Smith W.S., Holmes S.E., Flavell S.U., Flavell D.J. (2019). A Flow cytometric method to quantify the endosomal escape of a protein toxin to the cytosol of target cells. Pharm. Res..

[21] Klein E., Klein G., Nadkarni J.S., Nadkarni J.J., Wigzell H., Clifford P. (1968). Surface IgM-kappa specificity on a Burkitt lymphoma cell in vivo and in derived culture lines. Cancer Res..

[22] Adams R.A., Flowers A., Davis B.J. (1968). Direct implantation and serial transplantation of human acute lymphoblastic leukemia in hamsters, SB-2. Cancer Res..

[23] Weng A., Görick C., Melzig M.F. (2009). A brief communication: Enhancement of toxicity of saporin-based toxins by *Gypsophila* Saponins–kinetic of the saponin. Exp. Biol. Med..

[24] Flavell D., Flavell S., Boehm D., Emery L., Noss A., Ling N., Richardson P., Hardie D., Wright D. (1995). Preclinical studies with the anti-CD19-saporin immunotoxin BU12-SAPORIN for the treatment of human-B-cell tumours. Br. J. Cancer.

[25] Scudiero D.A., Shoemaker R.H., Paull K.D., Monks A., Tierney S., Nofziger T.H., Currens M.J., Seniff D., Boyd M.R. (1988). Evaluation of a soluble tetrazolium/formazan assay for cell growth and drug sensitivity in culture using human and other tumor cell lines. Cancer Res..

[26] McMahon H.T., Boucrot E. (2011). Molecular mechanism and physiological functions of clathrin-mediated endocytosis. Nat. Rev. Mol. Cell Biol..

[27] Wang L.H., Rothberg K.G., Anderson R.G. (1993). Mis-assembly of clathrin lattices on endosomes reveals a regulatory switch for coated pit formation. J. Cell Biol..

[28] Lim J.P., Gleeson P.A. (2011). Macropinocytosis: An endocytic pathway for internalising large gulps. Immunol. Cell Biol..

[29] Rabinovitch M. (1995). Professional and non-professional phagocytes: An introduction. Trends Cell Biol..

[30] Bayer N., Schober D., Prchla E., Murphy R.F., Blaas D., Fuchs R. (1998). Effect of bafilomycin A1 and nocodazole on endocytic transport in HeLa cells: Implications for viral uncoating and infection. J. Virol..

[31] De Duve C., De Barsy T., Poole B., Tulkens P. (1974). Lysosomotropic agents. Biochem. Pharmacol..

[32] Bowman E.J., Siebers A., Altendorf K. (1988). Bafilomycins: A class of inhibitors of membrane ATPases from microorganisms, animal cells, and plant cells. Proc. Natl. Acad. Sci. USA.

[33] Schliwa M. (1982). Action of cytochalasin D on cytoskeletal networks. J. Cell Biol..

[34] Granger E., McNee G., Allan V., Woodman P. (2014). The role of the cytoskeleton and molecular motors in endosomal dynamics. Semin. Cell Dev. Biol..

[35] Marshak D.R., Lukas T.J., Watterson D.M. (1985). Drug-protein interactions: Binding of chlorpromazine to calmodulin, calmodulin fragments, and related calcium binding proteins. Biochemistry.

[36] Al Soraj M., He L., Peynshaert K., Cousaert J., Vercauteren D., Braeckmans K., De Smedt S.C., Jones A.T. (2012). siRNA and pharmaco-logical inhibition of endocytic pathways to characterize the differential role of macropinocytosis and the actin cytoskeleton on cellular uptake of dextran and cationic cell penetrating peptides octaarginine (R8) and HIV-Tat. J. Control. Release.

[37] Ivanov A.I. (2008). Pharmacological inhibition of endocytic pathways: Is it specific enough to be useful?. Methods Mol. Biol..

[38] Ogiso T., Iwaki M., Mori K. (1981). Fluidity of human erythrocyte membrane and effect of chlorpromazine on fluidity and phase sep-aration of membrane. Biochim. Biophys. Acta.

[39] Murata T., Maruoka N., Omata N., Takashima Y., Fujibayashi Y., Yonekura Y., Wada Y. (2006). A comparative study of the plasma membrane permeabilization and fluidization induced by antipsychotic drugs in the rat brain. Int. J. Neuropsychopharmacol..

[40] Giocondi M.-C., Mamdouh Z., Le Grimellec C. (1995). Benzyl alcohol differently affects fluid phase endocytosis and exocytosis in renal epithelial cells. Biochim. Biophys. Acta Biomembr..

[41] Walenga R.W., Opas E.E., Feinstein M.B. (1981). Differential effects of calmodulin antagonists on phospholipases A2 and C in throm-bin-stimulated platelets. J. Biol. Chem..

[42] Wells A., Ware M.F., Allen F.D., Lauffenburger D.A. (1999). Shaping up for shipping out: PLCγ signaling of morphology changes in EGF-stimulated fibroblast migration. Cell Motil. Cytoskelet..

[43] Amyere M., Payrastre B., Krause U., Smissen P.V.D., Veithen A., Courtoy P.J. (2000). Constitutive macropinocytosis in onco-gene-transformed fibroblasts depends on sequential permanent activation of phosphoinositide 3-kinase and phospholipase C. Mol. Biol. Cell.

[44] Masson M., Spezzatti B., Chapman J., Battisti C., Baumann N. (1992). Calmodulin antagonists chlorpromazine and W-7 inhibit exoge-nous cholesterol esterification and sphingomyelinase activity in human skin fibroblast cultures. Similarities between drug-induced and Niemann-Pick type C lipidoses. J. Neurosci. Res..

[45] Lange Y., Ye J., Steck T.L. (2012). Activation mobilizes the cholesterol in the late endosomes-lysosomes of niemann pick Type C Cells. PLoS ONE.

[46] Smith W.S., Baker E.J., Holmes S.E., Koster G., Hunt A.N., Johnston D.A., Flavell S.U., Flavell D.J. (2017). Membrane cholesterol is essential for triterpenoid saponin augmentation of a saporin-based immunotoxin directed against CD19 on human lymphoma cells. Biochim. Biophys. Acta Biomembr..

[47] Koivusalo M., Welch C., Hayashi H., Scott C.C., Kim M., Alexander T., Touret N., Hahn K.M., Grinstein S. (2010). Amiloride inhibits macropinocytosis by lowering submembranous pH and preventing Rac1 and Cdc42 signaling. J. Cell Biol..

[48] Lagana A., Vadnais J., Le P.U., Nguyen T.N., Laprade R., Nabi I.R., Noël J. (2000). Regulation of the formation of tumor cell pseudopodia by the Na (+)/H (+) exchanger NHE1. J. Cell Sci..

[49] Boucrot E., Saffarian S., Massol R., Kirchhausen T., Ehrlich M. (2006). Role of lipids and actin in the formation of clathrin-coated pits. Exp. Cell Res..

[50] Fujimoto L.M., Roth R., Heuser J.E., Schmid S.L. (2000). Actin assembly plays a variable, but not obligatory role in receptor-mediated endocytosis in mammalian cells. Traffic.

[51] Schober D., Huber M., Bayer N., Murphy R.F., Fuchs R. (2005). Transferrin recycling and dextran transport to lysosomes is differentially affected by bafilomycin, nocodazole, and low temperature. Cell Tissue Res..

[52] Weng A., Bachran C., Fuchs H., Melzig M. (2008). Soapwort saponins trigger clathrin-mediated endocytosis of saporin, a type I ribosome-inactivating protein. Chem. Interact..

[53] Mellman I., Fuchs R., Helenius A. (1986). Acidification of the endocytic and exocytic pathways. Annu. Rev. Biochem..

[54] Redmann M., Benavides G.A., Berryhill T.F., Wani W.Y., Ouyang X., Johnson M.S., Ravi S., Barnes S., Darley-Usmar V.M., Zhang J. (2017). Inhibition of autophagy with bafilomycin and chloroquine decreases mitochondrial quality and bioenergetic function in primary neurons. Redox Biol..

[55] Yuan N., Song L., Zhang S., Lin W., Cao Y., Xu F., Fang Y., Wang Z., Zhang H., Li X. (2015). Bafilomycin A1 targets both autophagy and apoptosis pathways in pediatric B-cell acute lympho-blastic leukemia. Haematologica.

[56] Polito L., Bortolotti M., Mercatelli D., Battelli M.G., Bolognesi A. (2013). Saporin-S6: A useful tool in cancer therapy. Toxins.

[57] Lippincott-Schwartz J., Fambrough D.M. (1987). Cycling of the integral membrane glycoprotein, LEP100, between plasma membrane and lysosomes: Kinetic and morphological analysis. Cell.

[58] Chapman R.E., Munro S. (1994). Retrieval of TGN proteins from the cell surface requires endosomal acidification. EMBO J..

