# Supplementary material for: The Effect of Small Molecule Pharmacological Agents on the Triterpenoid Saponin Induced Endolysosomal Escape of Saporin and a Saporin-Based Immunotoxin in Target Human Lymphoma Cells
Source: Biomedicines. 2021 Mar 15;9(3):300. doi: 10.3390/biomedicines9030300 (PMC8000476; doi:10.3390/biomedicines9030300)
Supplement: Supplementary file 1 [file biomedicines-09-00300-s001.zip › Supplemental Figures Legends.docx]

**Figure S1. Investigation of the Inhibition of SA Mediated Endolysosomal Escape of SAP-AF in HSB-2 Cells by Pharmacological Agents as Measured by Pulse Width Analysis**

The effect of chlorpromazine (A), EIPA (B), cytochalasin D (C), nocadazole (D), chloroquine (E) and bafilomycin A1 (F) on the SA mediated endolysosomal escape of SAP-AF in HSB-2 cells. Charts show the changes in FITC-W over time in cells treated with 1 µg/ml of SA in the presence (▽) and absence (○) of inhibitor. Untreated control cells are shown in each chart for comparison in the presence (▼) and absence (●) of inhibitor. Each datum point represents the calculated mean of three experiments each performed in duplicate and error bars one standard deviation either side of this mean.

**Figure S2. Investigation of the Inhibition of SA Mediated Endolysosomal Escape of OKSAP-AF in HSB-2 Cells by Pharmacological Agents as Measured by Pulse Width Analysis**

The effect of chlorpromazine (A), EIPA (B), cytochalasin D (C), nocadazole (D), chloroquine (E) and bafilomycin A1 (F) on the SA mediated endolysosomal escape of SAP-AF in HSB-2 cells. Charts show the changes in FITC-W over time in cells treated with 1 µg/ml of SA in the presence (▽) and absence (○) of inhibitor. Untreated control cells are shown in each chart for comparison in the presence (▼) and absence (●) of inhibitor. Each datum point represents the calculated mean of three experiments each performed in duplicate and error bars one standard deviation either side of this mean.

**Figure S3. Effect of Cytochalasin D on Daudi Cell Morphology**

Dot plots showing the forward (FSC) and side scatter (SSC) of Daudi cells, both untreated controls (A) and cells treated with 0.75 µM cytochalasin D for 48 hours (B) as recorded by flow cytometry. An overlay of these plots is presented in (C).

**Figure S4. Confocal Microscopy Showing the Effect of Bafilomycin A1 on Daudi Cells Containing OKSAP-AF**

Confocal microscopy showing Daudi Cells preloaded with OKSAP-AF (Green) for 24 hours followed by incubation in the absence (A) and presence (B) of bafilomycin A1. The nucleus was stained with Hoechst 33342 (red). Images presented are maximum projections of 21 x 1 µm Z-stacks. Scale bar represents 10 µm.

**Figure S5. Inhibition of SA-Mediated Augmentation of Saporin Cytotoxicity in HSB-2 Cells by the Various Pharmacological Agents Studied**

Effect of chlorpromazine (A), EIPA (B), nocodazole (C), cytochalasin D (D), chloroquine A1 (E) and bafilomycin A1 (F) on the cytotoxicity of saporin and saporin used in combination with 1 µg/ml of SA. A-F: Dose-response curves determined by XTT assay for saporin on HSB-2 cells with each agent in the absence (▼) and presence (▽) of SA. In each chart the data for saporin without each agent in the absence (●) and presence (○) of SA is also presented for comparison. Each datum point represents the calculated mean of four experiments each performed in quadruplicate cell cultures and the error bars one standard deviation either side of this mean. The EC_50_ obtained from each curve is shown against the perpendicular dotted line. G: Fold increases in saporin cytotoxicity with 1µg/ml SA in control (●), chlorpromazine (■), EIPA (▲), nocodazole (▼), cytochalasin D (◆), chloroquine (○) and bafilomycin A (□). Dots represent fold increase for individual experiments with the lines showing the mean and one standard deviation either side of this mean. Augmentation was significantly abrogated by chlorpromazine (p = 0.0286 *), EIPA (p = 0.0286 *), cytochalasin D (p = 0.0286 *), chloroquine (p = 0.0286 *) and bafilomycin A1 (p = 0.0286 *) as determined by Mann-Whitney U-Test.

**Figure S6. Inhibition of SA-Mediated Augmentation of OKT10-SAP Cytotoxicity in HSB-2 Cells by the Various Pharmacological Agents Studied**

Effect of chlorpromazine (A), EIPA (B), nocodazole (C), cytochalasin D (D), chloroquine (E) and bafilomycin A1 (F) on the cytotoxicity of OKT10-SAP and OKT10-SAP used in combination with 1µg/ml of SA. A-F: Dose-response curves determined by XTT assay for OKT10-SAP on HSB-2 cells with each agent in the absence (▼) and presence (▽) of SA. In each chart the data for OKT10-SAP without inhibitor in the absence (●) and presence (○) of SA is also presented for comparison. Each datum point represents the calculated mean of four separate experiments each performed in quadruplicate cell cultures. Error bars represent one standard deviation either side of this mean. The EC_50_ obtained from each curve is shown against the perpendicular dotted line.
