# Supplementary figures and images for: The Effect of Small Molecule Pharmacological Agents on the Triterpenoid Saponin Induced Endolysosomal Escape of Saporin and a Saporin-Based Immunotoxin in Target Human Lymphoma Cells
Source: Biomedicines. 2021 Mar 15;9(3):300. doi: 10.3390/biomedicines9030300 (PMC8000476; doi:10.3390/biomedicines9030300)

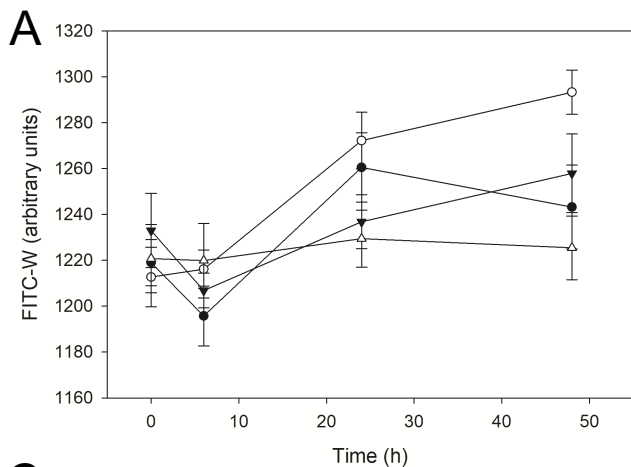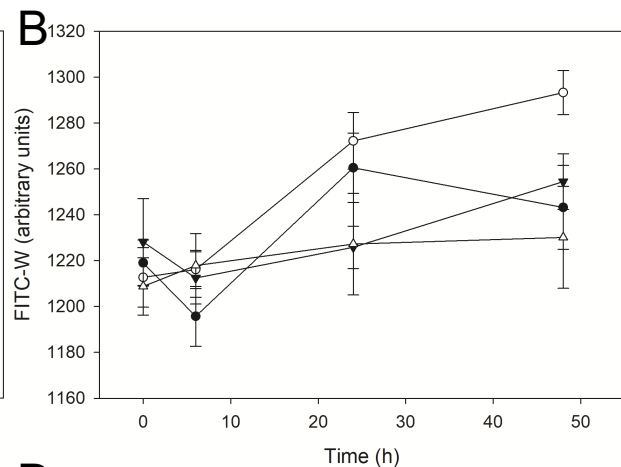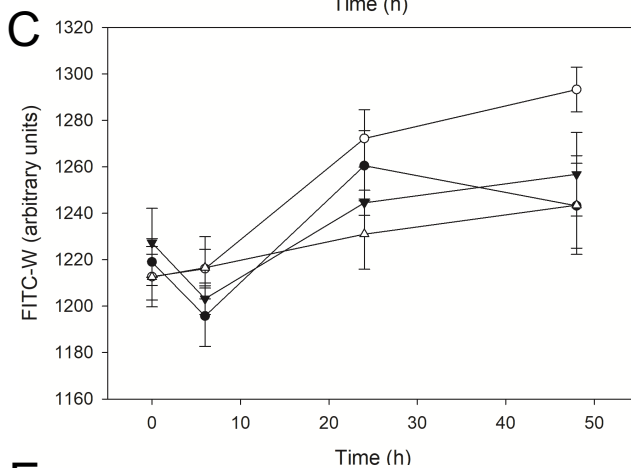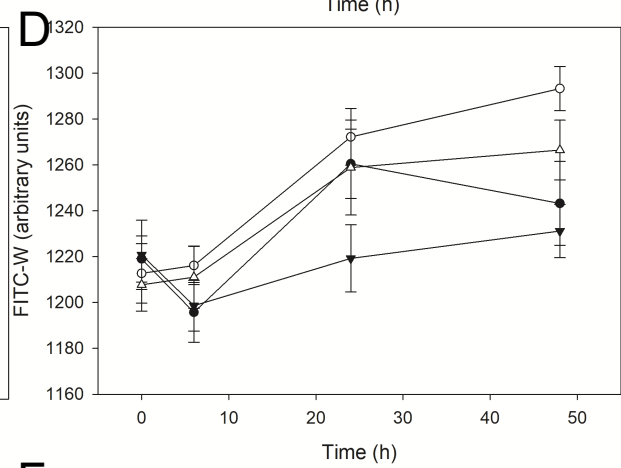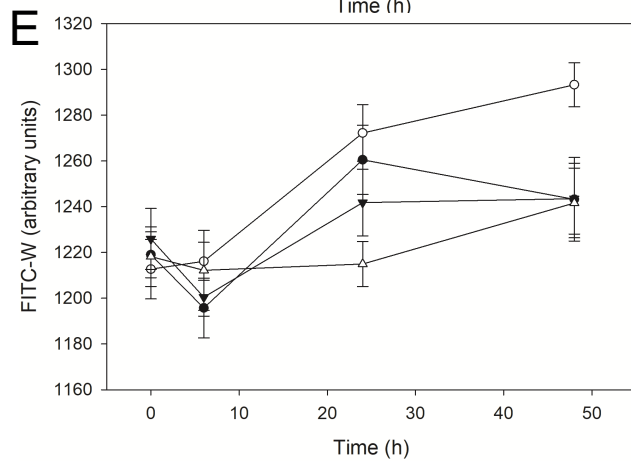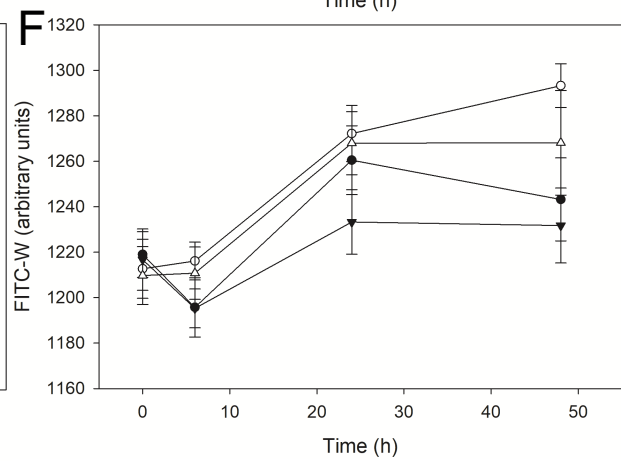

Supplement: Supplementary file 1 [file biomedicines-09-00300-s001.zip › Figure S1.pdf]

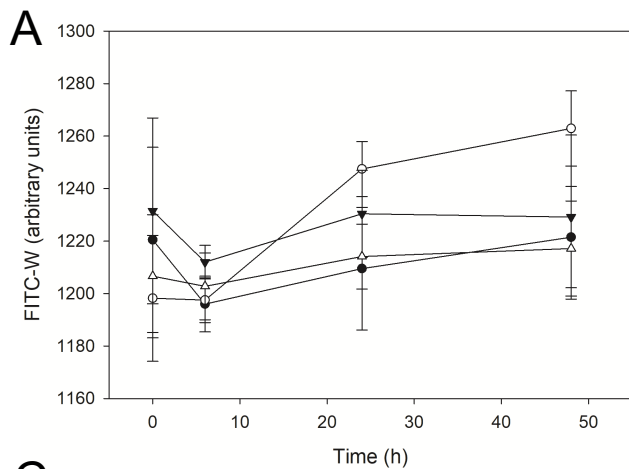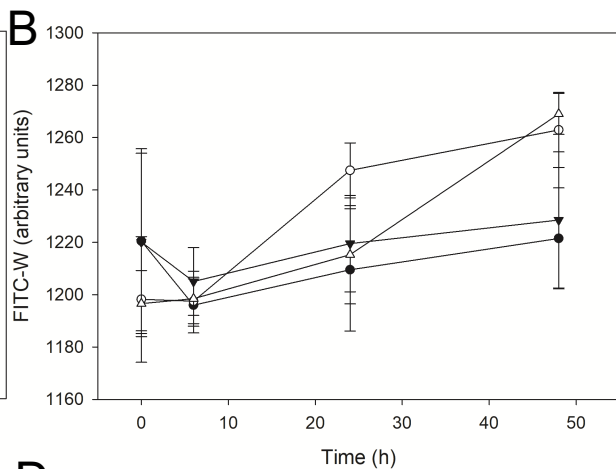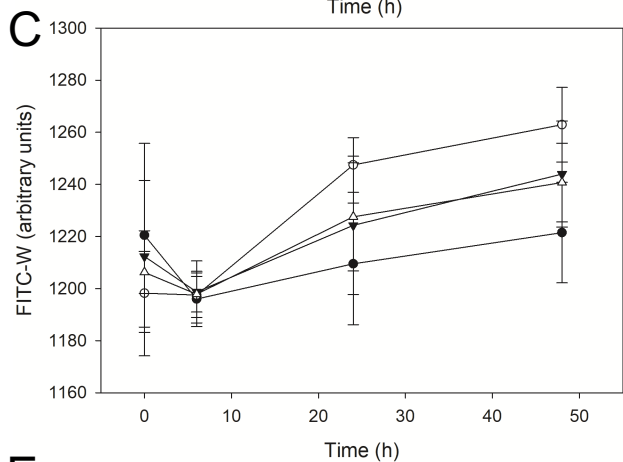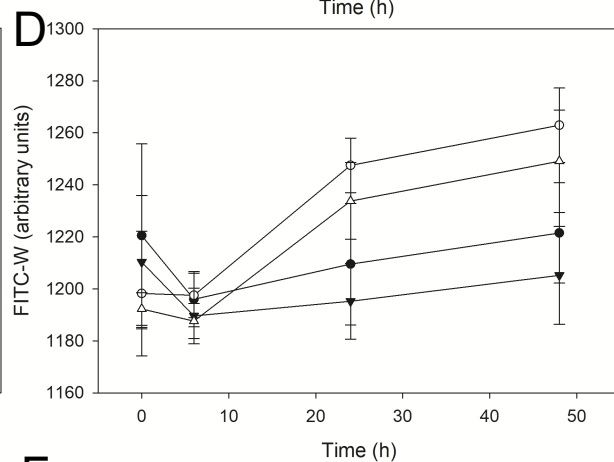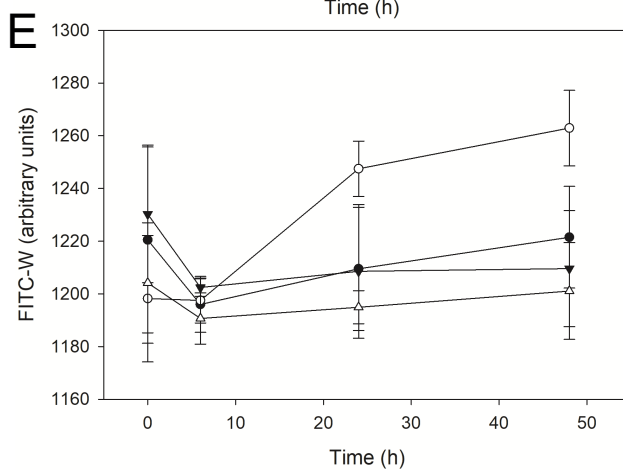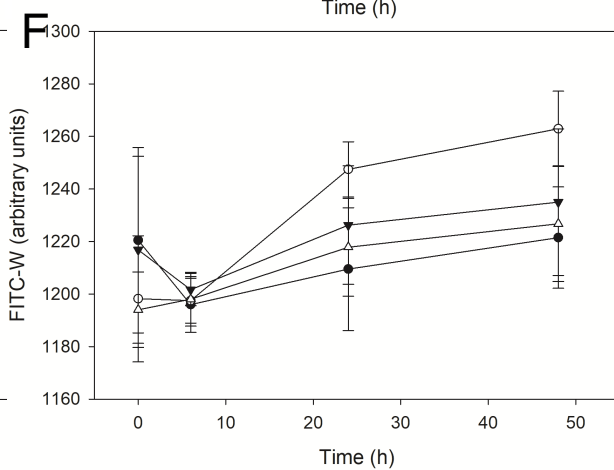

Supplement: Supplementary file 1 [file biomedicines-09-00300-s001.zip › Figure S2.pdf]

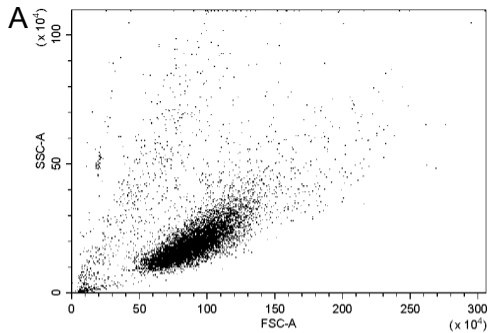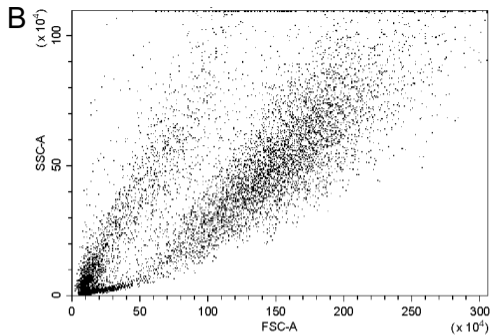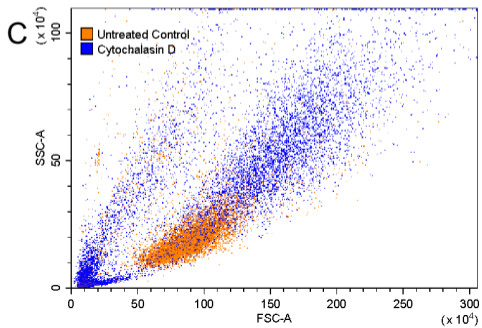

Supplement: Supplementary file 1 [file biomedicines-09-00300-s001.zip › Figure S3.pdf]

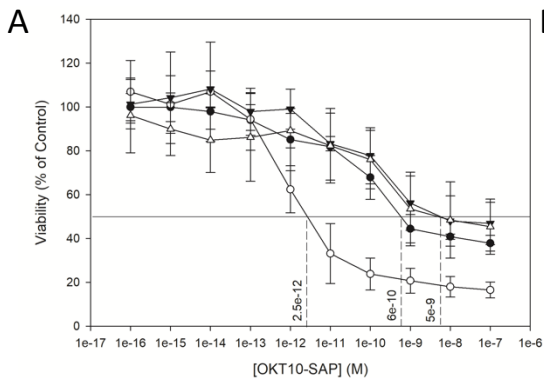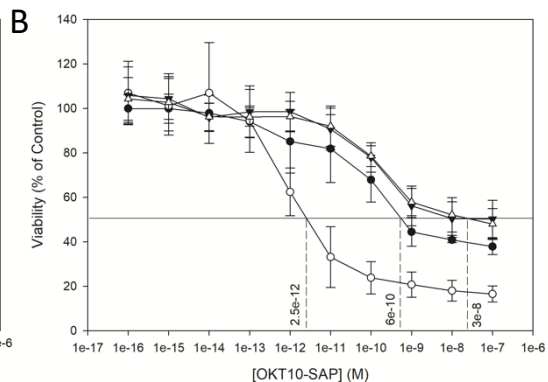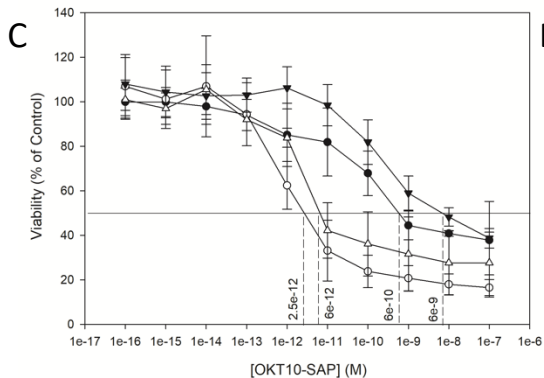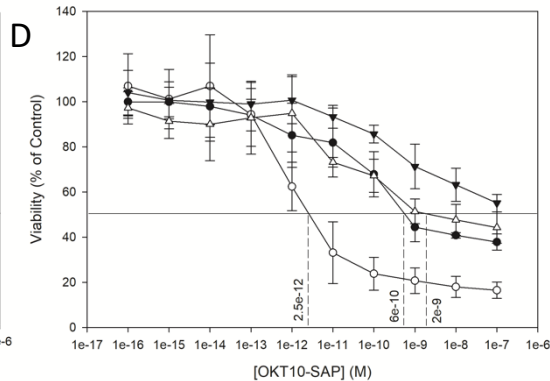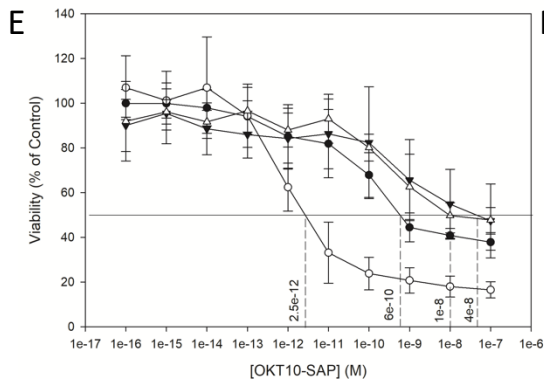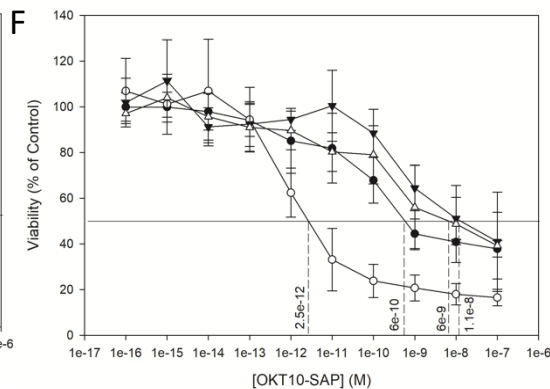

Supplement: Supplementary file 1 [file biomedicines-09-00300-s001.zip › Figure S6.pdf]

A

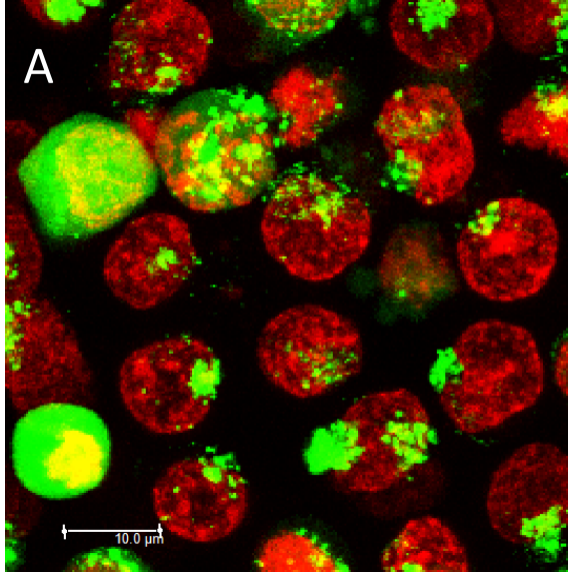

B

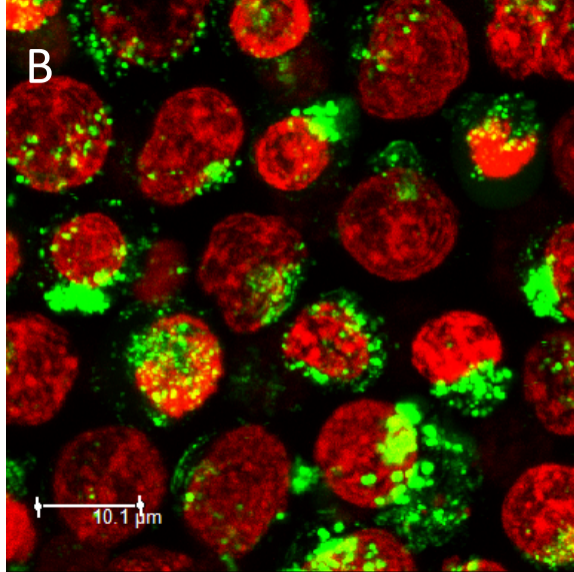

Supplement: Supplementary file 1 [file biomedicines-09-00300-s001.zip › Figure S4.pdf]

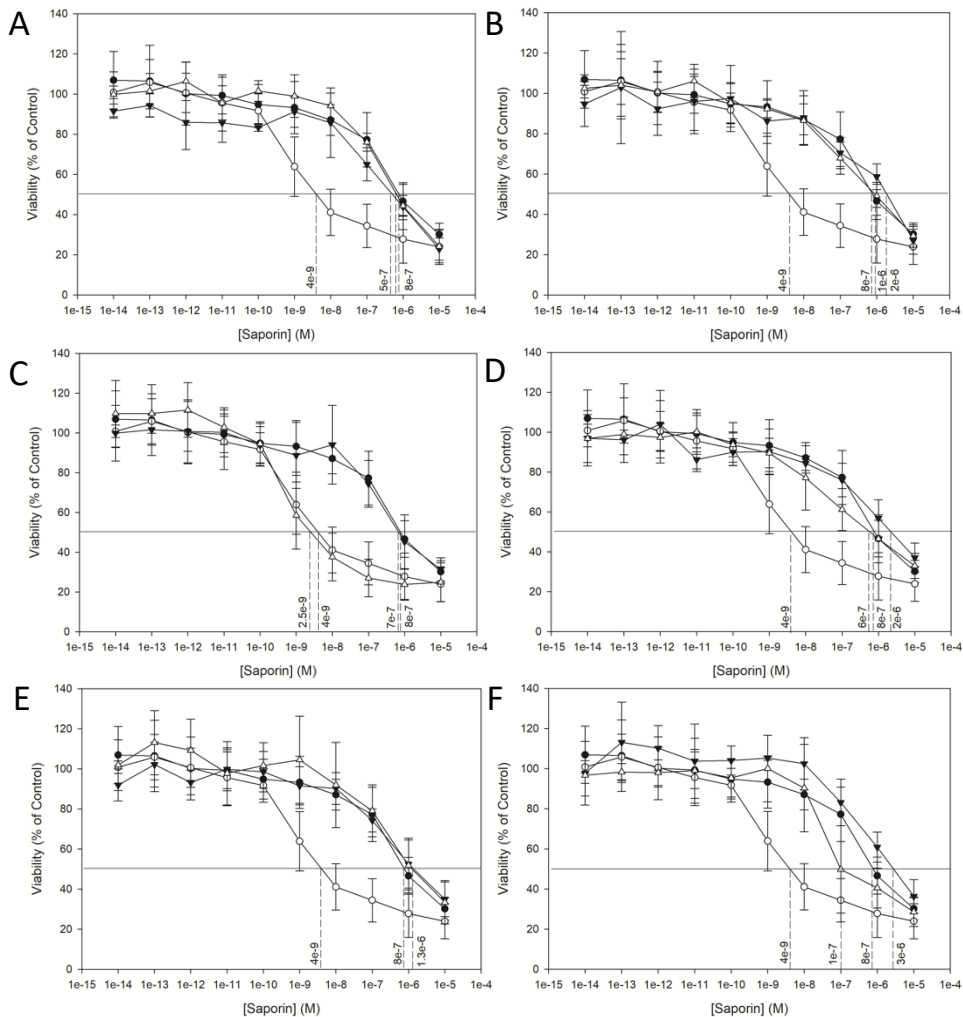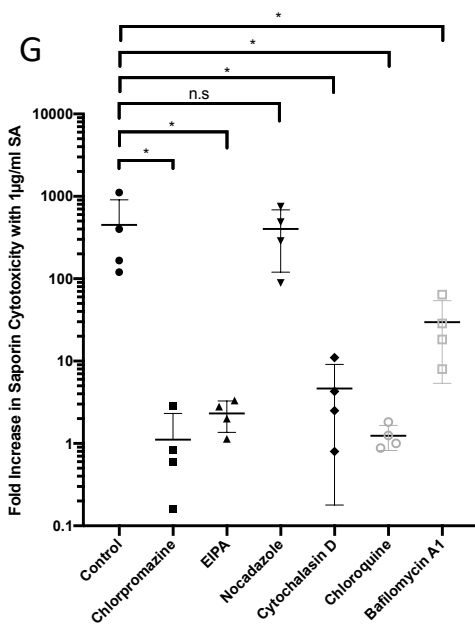

Supplement: Supplementary file 1 [file biomedicines-09-00300-s001.zip › Figure S5.pdf]
